# Treatment outcomes of standard (high dose) cisplatin and non‐standard chemotherapy for locally advanced head and neck cancer

**DOI:** 10.1002/cnr2.1674

**Published:** 2022-07-06

**Authors:** Muhammad Alamgeer, Andrew Coleman, Lachlan McDowell, Charles Giddings, Adnan Safdar, Elizabeth Sigston, Yang Wang, Ashwin Subramaniam

**Affiliations:** ^1^ Department of Medical Oncology Monash Health Clayton Victoria Australia; ^2^ Faculty of Medicine, Nursing and Health Sciences Monash University Clayton Victoria Australia; ^3^ Department of Radiation Oncology Peter MacCallum Cancer Centre Parkville Victoria Australia; ^4^ Department of Otolaryngology, Head and Neck Surgery Monash Health Clayton Victoria Australia; ^5^ Department of Surgery, School of Clinical Sciences at Monash Health, Faculty of Medicine, Nursing and Health Sciences Monash University Clayton Victoria Australia; ^6^ Department of Intensive Care Medicine Peninsula Health Frankston Victoria Australia; ^7^ Peninsula Clinical School Monash University Frankston Victoria Australia; ^8^ Australian and New Zealand Intensive Care Research Centre (ANZIC‐RC), School of Public Health and Preventive Medicine Monash University Melbourne Victoria Australia

**Keywords:** carboplatin/paclitaxel, cetuximab, cisplatin, locally advanced head and neck cancer, toxicity

## Abstract

**Introduction:**

Concurrent chemoradiotherapy with high‐dose (HD) cisplatin is the standard treatment for locally advanced head and neck squamous cell carcinoma (LA‐HNSCC). Due to the higher treatment‐related adverse effects with standard therapy, alternative regimens (non‐standard therapy), namely, lower dose weekly cisplatin, carboplatin/paclitaxel, or cetuximab are considered. There is, however, no consensus on non‐standard regimens. We aimed to investigate the efficacy and safety profile of these regimens.

**Methods:**

This single centre retrospective cohort study included all consecutive adult patients with newly diagnosed LA‐HNSCC treated with either standard or non‐standard regimens between January 2016 and April 2021. The primary outcome was 2‐year failure‐free survival (FFS). The secondary outcomes included acute toxicities, hospitalisation rates, dose modifications, treatment failure rates (TFR), and overall survival.

**Results:**

About 235 patients were included in the final analysis; median age was 61 years (IQR 55–67), and 87% were male. Most had oropharyngeal tumours (85.5%) and p16‐positivity was frequent (80%). About 56% received non‐standard regimens: weekly cisplatin = 79 and non‐cisplatin = 48. These patients had higher Charlson Comorbidity Index (CCI; *p* < .001) and lower European Cooperative Oncology Group (ECOG)‐0 (*p* = .003). There was no difference in 2‐year FFS (hazard ratio [HR] = 1.16; 95% confidence interval – [CI] 0.65–2.05), hospitalisation and grade‐3 toxicity rates between the two regimens. Nausea and vomiting were lower in the non‐standard regimen (3.0% vs. 16%, *p* < .001). Dose reductions, adjusted for age, sex, and CCI, were less likely in the non‐standard regimen (OR = 2.36; 95%‐CI: 1.01–5.49, *p* = .007).

**Conclusions:**

We demonstrated similar efficacy of lower dose weekly cisplatin and carboplatin/paclitaxel regimens and better safety profile of weekly cisplatin compared to standard HD cisplatin regimens for LA‐HNSCC. Multicenter randomised control trials are required in HD cisplatin‐ineligible patients.

## 1 INTRODUCTION

Squamous cell carcinoma of the head and neck (HNSCC) is the sixth most common cancer^1^ and continues to be a significant source of cancer‐related morbidity and mortality worldwide with close to half a million new cases and over 300 000 deaths yearly.^2^ While the most common risk factors for HNSCC worldwide remain heavy tobacco and alcohol use, there has been a dramatic rise in the incidence of human papillomavirus (HPV) mediated oropharyngeal cancer (OPC), particularly in the western countries.^3^, ^4^ This subset of HNSCC resulting from exposure to HPV type 16 and, less commonly, HPV‐18 is considered a distinct clinical and molecular entity and is more common in younger patients, usually without a history of heavy smoking.^5^ Most importantly, HPV‐related HNSCC is associated with a more favourable prognosis and possibly predicts a better therapeutic response than non‐HPV mediated disease.^6^

Patients with locally advanced disease (LA‐HNSCC), typically characterised by large tumours with local invasion, regional nodal metastases, or both,^6^, ^7^ are treated with a multi‐modality approach consisting of either definitive chemoradiotherapy (CRT), or surgery followed by radiotherapy (RT), or CRT in those with a high risk of recurrence.^8^, ^9^, ^10^, ^11^, ^12^ Several large randomised clinical trials have demonstrated a significant improvement in locoregional control and survival in both definitive and adjuvant settings with the addition of radio‐sensitising chemotherapy compared to RT alone. Based on these trials, the addition of cisplatin at 100 mg/m^2^ (HD‐cisplatin) administered once every 3 weeks concurrently with RT was established as a standard of care (SOC, Level I evidence).^9^ However, a high incidence of severe acute and late toxicities with HD cisplatin has led the investigation of alternative regimens with diminished treatment‐related complications and improved compliance. In the definitive setting, concurrent administration of the epidermal growth factor receptor (EGFR) antibody, cetuximab with RT demonstrated superior efficacy compared to RT alone.^13^ However, cetuximab failed to prove itself as an alternative to HD‐cisplatin in 3 recent large, randomised trials in low‐intermediate risk HPV‐positive OPCs.^14^, ^15^, ^16^ The addition of weekly, low‐dose (LD) cisplatin to RT has shown a more favourable toxicity profile without compromising efficacy in post‐operative treatment,^9^, ^17^ and better efficacy in low‐risk HPV positive OPC compared to cetuximab,^16^ but robust evidence for this regimen compared to HD cisplatin is still lacking in the definitive setting.^18^ Therefore, there is no established treatment consensus for patients who are ineligible for HD‐cisplatin, such as older or frailer individuals and those with absolute contraindications.

Although commonly used, the exact benefit of weekly LD‐cisplatin or non‐cisplatin regimens, such as carboplatin combined with paclitaxel (c/p) or fluorouracil (5FU), compared to HD‐cisplatin remains unknown in the definitive treatment of LA‐HNSCC. This study aimed to evaluate the efficacy and safety of SOC and non‐SOC regimens in all patients with LA‐HNSCC undergoing definitive chemoradiotherapy with a hypothesis that non‐SOC regimens are non‐inferior to SOC regimens.

## 2 MATERIALS AND METHODS

### 2.1 Ethics approval

This study was approved by the Monash Health Human Research Ethics Committee (ID: QA/67140/MonH‐2020‐224 253) with a waiver of informed consent.

### 2.2 Study design, setting, and patients

**FIGURE 1:**
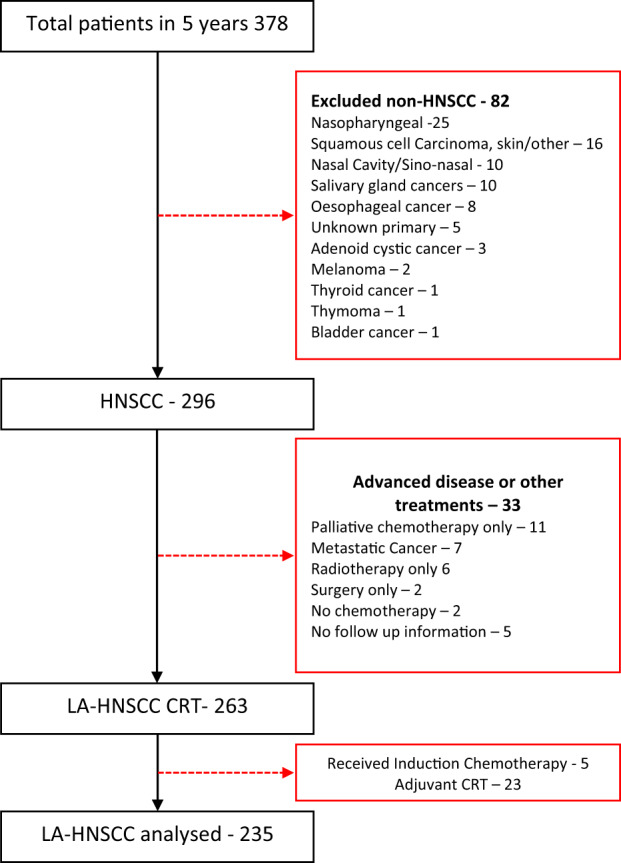
Flow diagram to demonstrate patient inclusion

We conducted a retrospective cohort study of all consecutive adult patients (aged ≥18 years) with newly diagnosed LA‐HNSCC (T3–T4, and/or N1‐3, according to the American Joint Council on Cancer [AJCC] 8th edition staging system for head and neck SCC)^19^ undergoing definitive CRT over a 5.4‐year period between January 2016 and April 2021 at our institution. The patients were included if they had a new diagnosis of an LA‐HNSCC of the oral cavity, oropharynx, hypopharynx or larynx and underwent definitive chemoradiation with curative intent. Patients were excluded if they had cancers of the nasopharynx, nasal cavity, paranasal sinuses, or salivary glands, or cutaneous primaries. Patients were also excluded if they had metastatic HNSCC, if they did not receive any chemotherapy or were treated with a palliative intent (Figure 1).

### 2.3 Choice of chemotherapy regimen

The treating clinicians determined the chemotherapy regimen. HD‐cisplatin (at a dose of 100 mg/m^2^, every 3 weeks for up to 3 doses) was commonly employed in definitive treatment regimens, whilst weekly LD‐cisplatin (40 mg/m^2^, every week for up to 7 doses) was commonly used as an alternative in situations where HD‐cisplatin was perceived to potentially result in significant toxicities that could compromise the completion of RT treatment, based on the treating clinician's clinical judgement. There was no difference in terms of antiemetic use and fluid replacement for both cisplatin schedules, which were according to the standard Australian practice guidelines.^20^ Baseline audiometry was performed in selected patients based on clinical suspicion. Non‐cisplatin regimen (weekly carboplatin; AUC 2) with paclitaxel (45 mg/m^2^) [c/p] were opted for based on patient comorbidities that precluded cisplatin altogether, such as pre‐existing renal or hearing impairment or both; or if there was any other absolute contraindication to cisplatin. Cetuximab (400 mg/m^2^ loading dose followed by 250 mg/m^2^ weekly dose given concurrently with RT) was preferred in patients with absolute contraindications to any chemotherapy, such as significant organ dysfunction or corticosteroid‐sparing in patients with comorbidities.

### 2.4 Data collection

Patients' electronic medical records were searched for data collection and follow up information. Patient characteristics included demographic details, smoking status, performance status as per the European Cooperative Oncology Group (ECOG) and baseline comorbidity as measured by the Charlson Comorbidity Index (CCI). The site of primary tumour was recorded as well as its staging, according to the AJCC TNM 8th edition. HPV status for oropharyngeal primaries was recorded using the standard p16 immunohistochemistry (IHC) scoring system.^21^ Chemotherapy was grouped into SOC HD‐cisplatin (3‐weekly) and non‐SOC (weekly LD‐cisplatin and non‐cisplatin [cetuximab or c/p]) with dose omissions or reductions and dose intensity also recorded. Safety data included rates of grade 3 or higher treatment‐related adverse events (TRAEs), as per common terminology criteria for adverse events (CTCAE) version 5.0,^22^ as well as details of unplanned hospital admissions occurring from the start of radiotherapy until 30 days following treatment completion. Information related to hospitalisation such as primary diagnosis, length of stay (LOS), chemotherapy regimen and frequency of admissions were also collected. For patients admitted more than once, the LOS was calculated as the total number of days spent in hospital across all admissions and the reasons for admission diagnosis for this group corresponded to their longest inpatient episode.

### 2.5 Definitions

Treatment failure (TF) was defined as persistence or recurrence of the same disease at either locoregional or distant sites. Time‐to‐event analyses included time to treatment failure (locoregional and/or distant). Failure free survival (FFS) was defined as the absence of relapse (locoregional or distant), non‐relapse mortality or addition of another systemic therapy for the same cancer. Overall survival (OS) was defined as the time from diagnosis to death from any cause. Disease‐specific survival (DSS), defined as time from diagnosis to death from HNSCC was also calculated.

### 2.6 Follow‐up

Patients were followed up for a median of 30.4 months (range 12–76 months).

### 2.7 Study outcomes

The primary outcome was 2‐year FFS comparing the SOC and non‐SOC groups, with an added focus across four commonly used chemotherapeutic regimens. Secondary outcomes included incidences of grade‐3 toxicities, hospitalisation rates, dose modifications, and OS comparison between treatment groups. Separate subgroup analysis of the P16+ OP cohort were also performed.

### 2.8 Statistical analysis

Descriptive statistics were used to summarise patient characteristics, treatment variables and safety. Patient characteristics, safety and survival outcomes were compared across chemotherapy groups (cisplatin vs. non‐cisplatin) and subgroups (HD‐cisplatin, weekly LD‐cisplatin, c/p, and cetuximab). The independent sample *t*‐test or the Mann–Whitney *U* test was used to compare two groups and the Kruskal–Wallis *H* test for data belonging to more than two groups involving a continuous dependent variable. The Chi‐square (*χ* ^2^) test of independence was used to test associations between categorical variables. Patients alive or lost to follow‐up were censored. The Kaplan–Meier method was used to calculate survival and the log‐rank test was used to compare survival curves. Logistic regression analysis was performed for outcomes of interest for non‐standard treatments in relation to HD‐cisplatin therapy, adjusting for confounders (namely age, sex, CCI, ECOG, and P16 status). All *p*‐values presented are two‐sided and threshold for statistical significance was set at *p* < .05. The cutoff date for the analysis was 30 April 2022. Statistical analysis was performed using IBM® SPSS Statistics for Windows, Version 26.0.

The study was conducted in accordance with the Declaration of Helsinki (as revised in 2014).^23^ We present the following article as per the STROBE reporting checklist.

## 3 RESULTS

During the study period, 378 patients were diagnosed with head and neck cancers. Of these, 235 patients with LA‐HNSCC were included in the final analysis (Figure 1). The patients' age ranged from 22 to 82 years, with a median (IQR) age of 61 (55, 65) years. Most patients were male (83%). One hundred and fifty‐nine patients (67.6%) were current or former smokers. All but two patients had an ECOG performance status of 0–1 (*n* = 233, 99%). The breakdown of tumour characteristics as well as patient demographics is summarised in Table 1.

**TABLE 1:** Baseline characteristics—SOC (HD‐cisplatin) versus non‐SOC (LD‐cisplatin + non‐cisplatin) therapy

Characteristics	Overall patients (*n* = 235)	HD‐cisplatin (*n* = 108)	Non‐SOC (*n* = 127)	*p*‐value
Age (years) median (Q1, Q3)	62 (55, 67)	60 (55, 64)	64 (58, 70)	.001
Male sex, *n* (%)	205 (87.2%)	96 (88.9%)	109 (85.8%)	.48
ECOG				
0	134 (55%)	71 (65.7%)	63 (49.6%)	**.042**
1	99 (42.1%)	36 (33.3%)	63 (49.6%)	
2+	2 (1.0%)	1 (1.1%)	1 (0.9)	
CCI, median (Q1, Q3)	4 (3, 5)	4 (3, 4)	4 (3, 5)	**<.001**
Smoker (including Ex), *n* (%)	159 (67.7%)	76 (70.4%)	83 (65.4%)	.41
Alcohol, *n* (%)	109 (46.4%)	52 (48.1%)	57 (44.9%)	.62
TNM classification				
T0	8 (3%)	3 (3%)	5 (4%)	.81
T1/T2	124 (53%)	55 (51%)	69 (54%)	
T3/T3	103 (44%)	50 (46%)	53 (42%)	
N0	21 (9%)	11 (10%)	10 (8%)	.79
N1	131 (56%)	55 (51%)	76 (60%)	
N2	68 (29%)	35 (32%)	33 (26%)	
N3	15 (6%)	7 (7%)	8 (6%)	
Stage				
1	91 (35.8%)	37 (34.3%)	54 (42.5%)	.05
2	64 (23.0%)	34 (31.5%)	30 (23.6%)	
3	52 (22.1%)	29 (26.9%)	23 (18.1%)	
4	28 (9.8%)	8 (6.7%)	20 (11.4%)	
Primary cancer site				
Oropharynx	201 (85.5%)	95 (1.1%)	106 (83.5%)	.80
Larynx	13 (5.5%)	5 (4.6%)	8 (6.3%)	
Oral cavity	3 (1.2%)	1 (0.9%)	2 (1.5	
Hypopharynx/others	18 (7.6%)	7 (6.4%)	11 (8.6%)	
P16+	188 (80%)	90 (83.3%)	98 (77.2%)	.24

Abbreviations: CCI, Charlson Co‐morbidity Index; ECOG, European Cooperative Oncology Group; HD, high dose; LD, low‐dose (weekly); SOC, standard of care.

P values highlighted in ‘bold’ are statistically significant.

### 3.1 Treatment

#### 3.1.1 Chemotherapy

All patients were treated with definitive CRT (*n* = 183, 90%) treatment. The majority of tumours (*n* = 188, 80%) were HPV‐related (p16+) and 201 (85%) were of oropharyngeal origin. One hundred and eight patients (46%) received HD‐cisplatin while 127 (54%) received non‐SOC regimens. Within the non‐SOC group, the majority (*n* = 79, 62%) received LD cisplatin, 30 (23%) received c/p while 19 (15%) had cetuximab based treatment (Table S1). As expected, patients receiving HD‐cisplatin were more likely to be medically fit, with more patients having an ECOG performance status of 0 and lower median (and IQR) CCI score when compared to the non‐SOC group (Table 1). Age, cancer stage, primary site, smoking, and alcohol status were similar between the treatment arms.

The median cumulative cisplatin dose was 279 mg/m^2^ (100–300 mg/m^2^) for those treated with the HD‐cisplatin regimen and 280 mg/m^2^ (238–280 mg/m^2^) for the weekly LD‐cisplatin regimen. The majority patients treated with HD or weekly LD‐cisplatin regimens achieved a cumulative dose ≥200 mg/m^2^ (92.2% vs. 87.8%, *p* = .49). The dose intensity and modifications are summarised in Table S5.

#### 3.1.2 Radiotherapy

All patients were treated with highly conformal RT, either intensity modulated radiotherapy or volumetric arc therapy. Patients received a radical dose of 70 Gy in 35 fractions, concurrently with chemotherapy. All patients completed the planned doses of radiotherapy, except for one patient (<1%) in the definitive treatment group, who died from an unrelated medical cause.

### 3.2 Treatment efficacy

#### 3.2.1 Primary outcome

**FIGURE 2:**
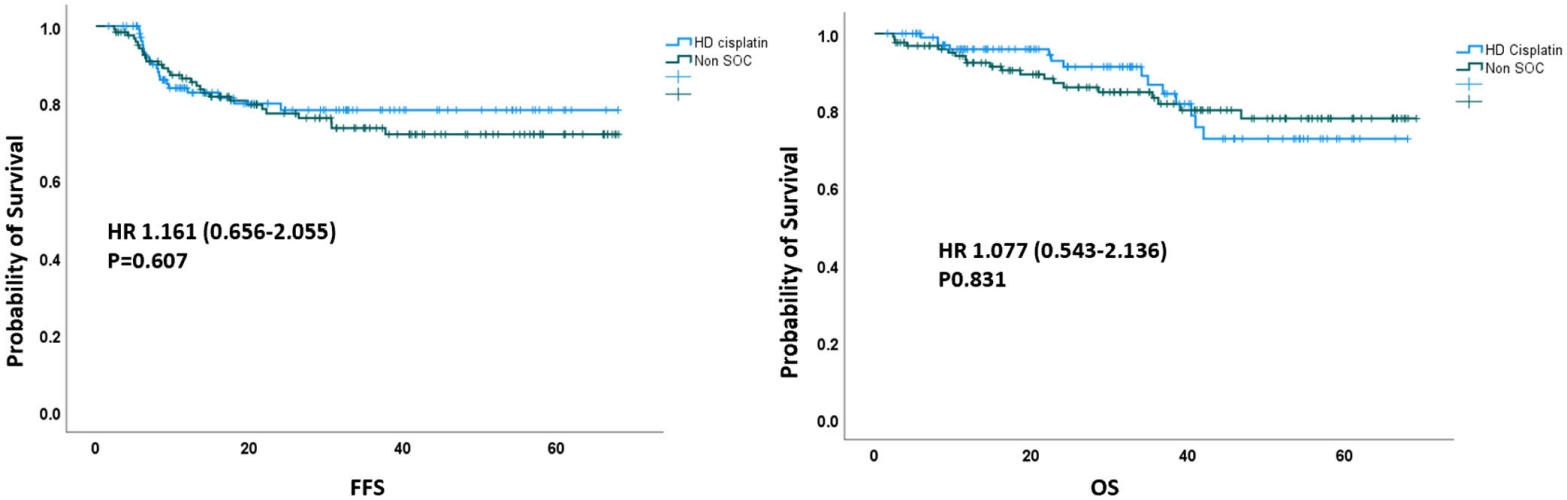
Primary outcome—failure free survival (FFS) and overall survival (OS) amongst the treatment groups

All patients were followed up for a median of 30.4 months (range 12–76 months). Forty‐one (17.4%) patients experienced treatment failure: 15 (6.4%) locoregional, and 32 (13.6%) distant. The 2‐year FFS rates were similar between the HD‐cisplatin and non‐SOC treatment groups (93.5% vs. 91.0%; HR: 1.61; 95% confidence interval [CI] 0.65–2.05, *p* = .60) (Figure 2). The 2‐year FFS was statistically not different between the various chemotherapy subgroups: 93.5% for HD‐cisplatin, 92.4% in weekly LD‐cisplatin, 87% in c/p and 92% in cetuximab regimens (Figure S1). In the entire cohort, 8 (3%) patients required salvage neck surgery due to persistent uptake on the follow‐up PET scans, and 3 (1.2%) had a viable residual tumour on histopathological analysis.

#### 3.2.2 Secondary outcomes

##### Treatment failure rates (TFR)

Two years TFR were similar, amongst the treatment groups (6.4% vs. 8.6%; *p* = .53) (Table 2).

**TABLE 2:** Toxicity outcomes amongst treatment groups

Outcomes	Overall patients (*n* = 235)	HD‐cisplatin (*n* = 108)	Non‐SOC (*n* = 127)	*p*‐value
Grade 3 toxicities	100 (42.6%)	42 (38.9%)	58 (45.7%)	.30
Mucositis	67 (28.5%)	28 (25.9%)	39 (30.7%)	.44
Febrile neutropenia	27 (11.5%)	14 (13%)	13 (10.2%)	.51
Nausea and vomiting	19 (16.2%)	16 (14.8%)	3 (2.4%)	**<.001**
Other				
Hearing impairment needing intervention	33 (14%)	24 (22.2%)	9 (7.1%)	**<.001**
Sepsis	16 (6.8%)	4 (3.7%)	12 (9.4%)	.32
Rash	9 (4.4%)	1 (1.1%)	8 (3.9%)	**.041**
Cytopenia^a^	38 (15.9%)	22 (20%)	16 (12.5%)	.10
Fever	5 (2.1%)	1 (0.9%)	4 (3.2%)	.17
Malnutrition	6 (2.9%)	3 (3.3%)	3 (1.5%)	.77
Acute kidney injury	7 (2.5%)	6 (4.6%)	1 (0.7)	.023
Electrolyte imbalance	4 (1.9%)	1 (1.1%)	3 (1.5%)	.44
VTE^b^	2 (0.9%)	1 (1.1%)	1 (0.9%)	.87
Miscellaneous	5 (2.4%)	3^d^ (3.3%)	2^e^ (1.8%)	.47
Unplanned hospitalisation within 30 days of treatment completion	79 (33.6%)	34 (31.5%)	45 (35.4%)	.52
Required salvage surgery	8 (3.4%)	4 (3.7%)	4 (3.1)	.50
Locoregional relapse	15 (6.4%)	6 (5.6%)	9 (7.1%)	.63
Distant relapse/metastasis	32 (13.6%)	15 (13.9%)	17 (13.4%)	.91
Mortality	31 (13.2%)	13 (12%)	18 (14.2%)	.63
2‐Year TFR (95%‐CI)	7.3% (5.8–15.9)	6.4% (3.2–18.8)	8.6% (5.2–17.2)	.45^c^

Abbreviations: HD, high dose; SOC, standard of care; TFR, treatment failure rates; TRAE, treatment‐related adverse events; VTE, venous thromboembolic disease.

^a^ Includes anaemia, thrombocytopenia, leukopenia, neutropenia, and febrile neutropenia.

^b^ One each of lower limb deep venous thrombosis and pulmonary embolism.

^c^ Log rank (Mantel‐Cox).

^d^ Two patients had fatigue, one had steroid‐related side effects.

^e^ One each of lymphoedema and paclitaxel‐related reaction.

P values highlighted in ‘bold’ are statistically significant.

##### Mortality

The overall mortality rate was 13.2% (*n* = 31), similar in the SOC (12%) and non‐SOC groups (14.2%); *p* = .63. Within the non‐SOC group, mortality was highest in the non‐cisplatin group (25%), and lowest is the weekly LD‐cisplatin group (7.7%) (Tables S2–S4). Importantly, most deaths (*n* = 5, 62%) in the non‐cisplatin group, were not cancer related, and the disease specific mortality was similar in the SOC (11%) and 7.8% in non‐SOC groups (6%, 10%, and 11%, respectively in the weekly, c/p and cetuximab groups) (Figure S1).

#### 3.2.3 Safety profile

##### Toxicity

A total of 100 (42%) patients experienced at least one TRAE of grade 3 severity, accounting for a total of 139 (59.1%) separate TRAEs (Table 2). Common acute grade 3 toxicities included mucositis in 67 (27.9%), febrile neutropaenia in 27 (11.5%), and nausea and vomiting in 19 (8%) patients. There were no treatment‐attributable grade 4 or 5 toxicities in our cohort. Clinically significant hearing impairment or severe tinnitus requiring intervention, such as drug cessation or treatment change and audiology referrals, occurred in 33 (14.2%) patients; this was significantly higher in the SOC regimen (22%, *n* = 24) versus 7.1% (*n* = 9) when compared with the non‐SOC group (*p* = .003). Similarly, the SOC regimen caused higher rates of acute renal failure (4.6% vs. 0.7%, *p* = .023) and nausea and vomiting (14.8% vs. 2.4%, *p* < .001).

Amongst the non‐SOC group, the non‐cisplatin group had the highest rates of acute grade 3 toxicities overall (54.5%). Grade 3 skin rash was more common in cetuximab group (*n* = 8, 50%) (Tables S2–S4).

##### Hospitalisation

Overall, 33.6% of patients (*n* = 79) had unplanned hospital admissions within 30 days of radiotherapy completion. Common causes of hospitalisation included: composite trigger of oral mucositis, dysphagia, and malnutrition (40%, *n* = 25), infection and sepsis (30%, *n* = 17); febrile neutropenia (22%, *n* = 14); and nausea and vomiting (8%, *n* = 5). Hospitalisation rates were similar amongst the SOC (31.5%) and non‐SOC (35.4%) treatment groups (*p* = .52). Within the treatment subgroups, hospitalisation rates were significantly lower (26.6%) in the weekly LD‐cisplatin and higher in the non‐cisplatin (50%) (Table 2 and Tables S2–S4).

Efficacy and safety outcomes of interest for non‐SOC compared to SOC HD‐cisplatin (adjusted for age, sex, and CCI) are summarised in Table 3.

**TABLE 3:** Unadjusted and adjusted (for age, male sex, and Charlson Comorbidity Index) outcomes of interest for non‐standard treatments in relation to HD‐cisplatin therapy

Variables	Univariate analysis			Multivariate analysis		
	OR	95%‐CI	*p*‐value	OR	95%‐CI	*p*‐value
Dose reduction	2.66	1.19–5.97	**.018**	2.36	1.01–5.49	**.007**
Grade‐3 toxicity	1.32	0.78–2.22	.30	0.76	0.44–1.32	.32
Unplanned hospitalisation	0.84	0.49–1.44	.52	0.95	0.53–1.71	.87
Mortality	0.83	0.40–1.78	.63	1.01	0.43–2.37	.99
Failure‐free survival	0.89	0.44–1.71	.13	0.69	0.33–1.42	.31
Locoregional relapse	0.77	0.27–2.24	.63	0.82	0.25–2.69	.74
Distant relapse	1.04	0.49–2.20	.91	1.12	0.50–2.49	.79

Abbreviation: HD, high dose.

P values highlighted in ‘bold’ are statistically significant.

#### 3.2.4 Subgroup analysis: outcomes in P16+ OPC cohort

A total of 188 patients were P16+ OPCs. The majority (*n* = 144, 95%) were stage 1/2 and underwent definitive CRT. There were relatively more patients in the weekly LD‐cisplatin group (*p* = .05), but no significant difference was found in terms of efficacy (2‐year FFS and mortality) or safety profile amongst the subgroups (Table S6).

## 4 DISCUSSION

In this single centre retrospective cohort study of patients with LA‐HNSCC undergoing definitive chemoradiotherapy, clinical outcomes of non‐SOC regimens (weekly LD‐cisplatin, c/p, and cetuximab) were compared to the SOC HD‐cisplatin regimen. There was no difference in FFS and OS rates amongst the treatment groups; however, the outcomes in terms of toxicity and compliance were worse in the SOC HD‐cisplatin group. Weekly LD‐cisplatin resulted in a similar efficacy with a better safety profile, but the non‐cisplatin regimens had relatively poor clinical outcomes in terms of safety profiles, compared to the cisplatin groups.

Despite HD‐cisplatin being the accepted SOC for more than two decades,^9^ high rates of systemic and mucosal toxicities during CRT are well reported.^24^ Our findings were no different. We demonstrated high rates of toxicity and poor compliance with SOC HD‐cisplatin.

Contrary to the phase 3 randomised control trial that used a lower weekly dose of cisplatin (30 mg/m^2^) and underestimated the efficacy,^25^ our findings of improved therapeutic efficacy and safety profile with weekly LD‐cisplatin (40 mg/m^2^) were comparable to a recent Japanese phase 2/3 trial which showed non‐inferiority of weekly cisplatin (40 mg/m^2^) and a favourable safety profile, compared to HD‐cisplatin for post‐operative patients with high‐risk LA‐HNSCC.^17^ Our study findings warrant weekly LD‐cisplatin to be studied in a larger prospective phase 3 clinical trial in the non‐surgical, definitive treatment of LA‐HNSCC.

The anti‐epidermal‐growth factor receptor (EGFR) antibody, cetuximab showed benefit when combined with RT, compared to RT alone in a prospective phase 3 trial,^13^ but the benefit was limited to oropharyngeal SCC and only with a particular (concomitant boost) RT regimen. More recently, two large randomised trials have shown inferior outcomes with cetuximab compared to HD cisplatin when given in combination with RT.^14^, ^15^ Despite the low number of patients, our study also established that cetuximab, when used in patients with absolute contraindications to cisplatin, showed benefit when combined with RT in the treatment of patients with LA‐HNSCC and is still an acceptable option.

Another viable treatment option, supported by clinical research is carboplatin plus fluorouracil.^8^, ^26^ However, increased risk of oral mucositis, prolonged infusion times, and requirement of central venous access devices have made fluorouracil as a less favourable regimen. Based on limited evidence in HNSCC and extrapolated evidence from other malignancies, such as non‐small cell lung cancer and oesophageal cancers,^27^ carboplatin plus paclitaxel (c/p) appears to be a reasonable option in cisplatin‐ineligible patients. Paclitaxel containing regimen has shown activity when used as a neo‐adjuvant agent in the treatment of LA‐HNSCC,^28^ but the evidence is relatively sparse for the definitive treatment setting. In our study, the similar efficacy of non‐cisplatin regimens may warrant further investigation of c/p regimen in the definitive setting. Since the disease‐specific OS was similar in all cohorts, the numerically higher mortality rates and higher toxicities in c/p and cetuximab could perhaps be due to selection bias (more patients with advanced age and poor CCI at baseline in the non‐cisplatin group). Since cetuximab has already been proven inferior to cisplatin in the definitive treatment of HPV‐OPC,^14^, ^15^ our data indicate a possible similar efficacy of c/p regimen, supporting the need for further evaluation in a prospective cohort of cisplatin‐ineligible, but otherwise healthy patients.

Further novel strategies in cisplatin‐ineligible patients with LA‐HNSCC are already being investigated. Immune checkpoint inhibitors have shown promise in recurrent metastatic HNSCC, and are being studied in LA‐HNSCC. So far, pembrolizumab has failed to offer benefits over cetuximab^29^ and another agent (nivolumab) is currently being trialled for the cisplatin non‐eligible LA‐HNSCC population (NCT03349710).

Our study provides a contemporary pragmatic overview of LA‐HNSCC treatment in an Australian setting and highlights the challenges of appropriate chemotherapy selection for concurrent chemoradiation. This study has a few limitations that need to be acknowledged. First, the study was conducted in a single centre, and the results may have been influenced by unmeasured factors such as illness severity and hospital‐specific policies, procedures and resource capability that drive clinical decision‐making. Care must therefore be taken when generalising the results of this study to other healthcare institutions, particularly smaller communities, and rural hospitals. Second, the population was skewed in favour of cisplatin‐based regimens with some baseline differences in patient characteristics between the groups. However, this does reflect an accurate real‐world sample in countries with similar epidemiological spreads. Third, the short follow‐up could have resulted in the inability to capture late toxicities, as despite relatively high cure rates for patients with LA‐HNSCC, the late toxicity profile has been associated with a negative impact on quality of life.^30^

## 5 CONCLUSIONS

Our study demonstrates that weekly LD‐cisplatin has similar efficacy to HD cisplatin but is less toxic. Other “less toxic” regimens had higher toxicities than weekly LD‐cisplatin but possibly had similar efficacy. There is an urgent need for newer treatments in patients unable to receive cisplatin, and further randomised trials are required.

## AUTHOR CONTRIBUTIONS

*Conceptualization*, M.A., E.S.; *Data Curation*, M.A., A.C., C.G., L.M., A.S., Y.W., A.S.; *Formal Analysis*, M.A., A.C., Y.W.; *Methodology*, M.A., A.S.; *Project administration*, M.A.; *Supervision*, M.A., A.S.; *Writing – Original Draft*, M.A., L.M., A.S.; *Writing – Review and Editing*, M.A., A.C., C.G., L.M., A.S., E.S., Y.W., A.S.; *Software*, A.C., A.S.

## CONFLICT OF INTEREST

The authors have stated explicitly that there are no conflicts of interest in connection with this article.

## Supporting information


**Figure S1** FFS and OS comparison of all four treatment groups HD‐cisplatin, weekly cisplatin, carboplatin/paclitaxel (c/p), and cetuximab.
**Table S1** Baseline characteristics of Non‐SOC regimens
**Table S2** HD‐cisplatin‐based versus LD‐cisplatin‐based therapy
**Table S3** Non‐cisplatin‐based versus LD‐cisplatin‐based therapy
**Table S4** HD‐cisplatin‐based versus non‐cisplatin‐based therapy
**Table S5** Chemotherapy cumulative dose, intensity, and modifications
**Table S6** Analysis of outcomes in P16+ OP SCC cohort

## Data Availability

The data that support the findings of this study are available from the corresponding author upon reasonable request.
