# Supplementary material for: Treatment outcomes of standard (high dose) cisplatin and non‐standard chemotherapy for locally advanced head and neck cancer
Source: Cancer Rep (Hoboken). 2022 Jul 6;6(1):e1674. doi: 10.1002/cnr2.1674 (PMC9875652; doi:10.1002/cnr2.1674)
Supplement: Supplementary file 1 — Figure S1 FFS and OS comparison of all four treatment groups HD‐cisplatin, weekly cisplatin, carboplatin/paclitaxel (c/p), and cetuximab. Table S1 Baseline characteristics of Non‐SOC regimens Table S2 HD‐cisplatin‐based versus LD‐cisplatin‐based therapy Table S3 Non‐cisplatin‐based versus LD‐cisplatin‐based therapy Table S4 HD‐cisplatin‐based versus non‐cisplatin‐based therapy Table S5 Chemotherapy cumulative dose, intensity, and modifications Table S6 Analysis of outcomes in P16+ OP SCC cohort [file CNR2-6-e1674-s001.docx]

|  | **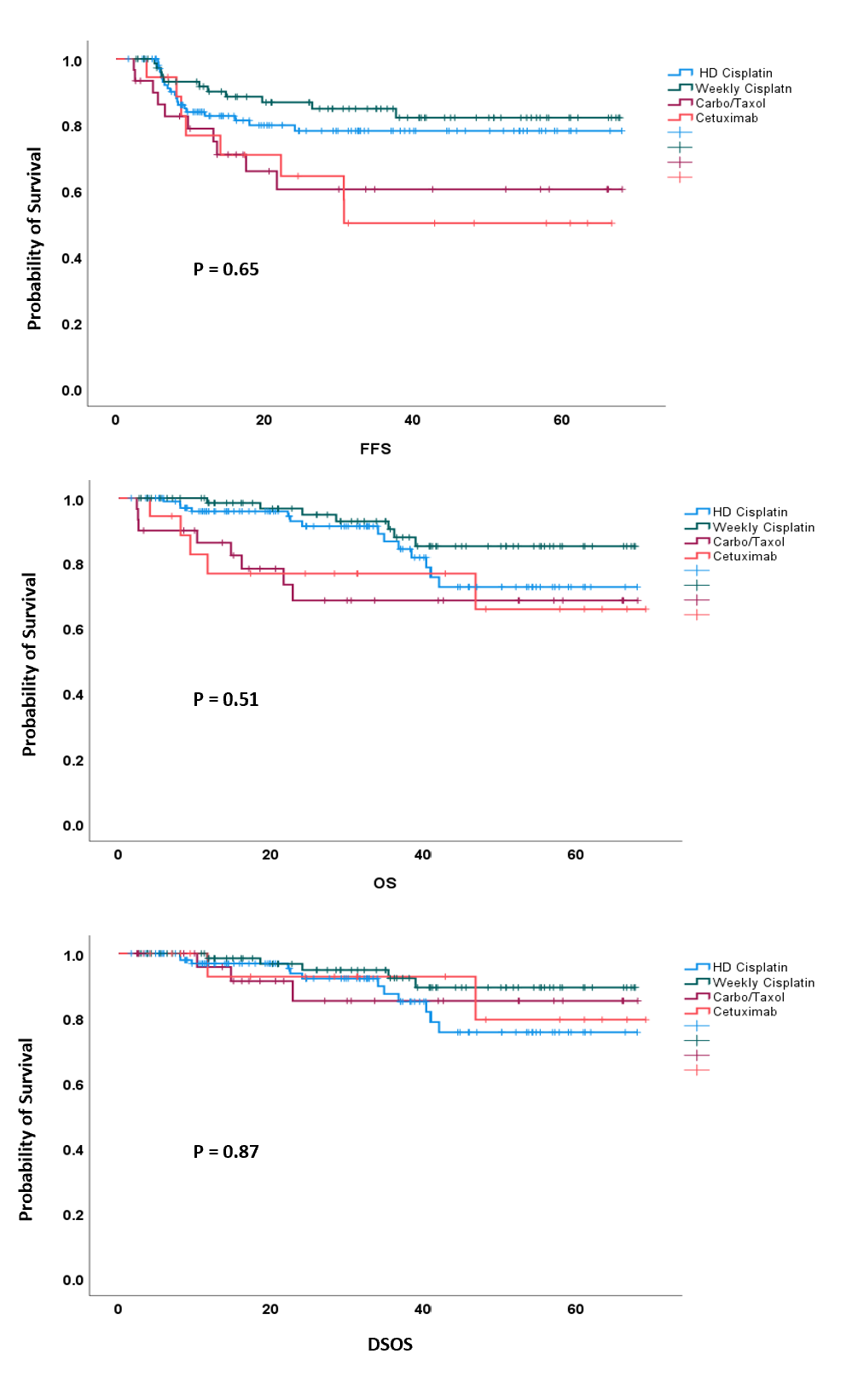**  **Supplementary Figure 1** : FFS and OS comparison of all 4 treatment groups HD-cisplatin, weekly cisplatin, carboplatin/paclitaxel (c/p), and cetuximab. |
| --- | --- |

**Supplementary Table 1:** Baseline Characteristics of Non-SOC regimens

| **Characteristics** | **Weekly Cisplatin (n=78)** | **Carboplatin/Paclitaxel  (n=30)** | **Cetuximab  (n=18)** | **p-value** |  |
| --- | --- | --- | --- | --- | --- |
| Age (years) Median (Q1, Q3) | 60 (54.8, 64) | 68.5 (61.3, 73.8) | 70 (66, 74.5) | **<0.001** |  |
| Male Sex, n (%) | 68 (87.2%) | 28 (93.3%) | 13 (72.2%) | <0.001 |  |
| ECOG | | | | | |
| 0 | 46 (59%) | 10 (33.3%) | 6 (3.3%) | **0.003** |  |
| 1 | 32 (41.0%) | 20 (66.7%) | 11 (61.1%) |  |  |
| 2+ | 0 (0) | 0 (0) | 1 (5.6%) |  |  |
| CCI, median (Q1, Q3) | 4 (3, 4) | 5 (4, 5) | 6 (5, 8) | **<0.001** |  |
| Smoker (including Ex), n (%) | 48 (61.5%) | 23 (76.7%) | 11 (61.1%) | 0.36 |  |
| Alcohol, n (%) | 34 (43.6%) | 15 (50%) | 7 (38.9%) | 0.79 |  |
| Stage | | | | | |
| 1 | 35 (44.9%) | 10 (33.3%) | 9 (50%) | 0.015 |  |
| 2 | 21 (26.9%) | 5 (16.7%) | 3 (16.7%) |  |  |
| 3 | 15 (19.20%) | 7 (23.3%) | 1 (5.6%) |  |  |
| 4 | 7 (9.0%) | 8 (26.7%) | 5 (27.8%) |  |  |
|  |  |  |  |  |  |
| Primary cancer site | | | | | |
| - Oropharynx | 68 (87.1%)1 (77.2%) | 25 (83.3%)1 (69.2%) | 13 (72.2%) | 0.13 |  |
| - Larynx | 6(7.6%) | 2 (6.6%) | 0(0%) |  |  |
| - Oral Cavity | 1 (1.2%)4 (5.4%) | 1 (3.3%) | 1 (5.5%) |  |  |
| - Hypopharynx/Other | 4 (5.1%) | 2 (6.6%) | 5 (27.7%) |  |  |
| - - P16+ | 63 (8.70%) | 22 (73.3%) | 13 (72.2%) | 0.50 |  |

**Supplementary Table 2:** HD-Cisplatin-based vs. LD-cisplatin-based therapy

| **Characteristics** | **Overall patients (n=187)** | **HD-Cisplatin  (n=108)** | **LD-Cisplatin  (n=79)** | **p-value** |
| --- | --- | --- | --- | --- |
| **Exposure** | | | | |
| Age (years), median (Q1, Q3) | 60 (54.8, 64) | 60 (54.8, 64) | 60 (54.8, 64) | 0.63 |
| Male Sex, n (%) | 164 (88%) | 96 (88.9%) | 68 (87.2%) | 0.16 |
| ECOG | | | | |
| - - 0 | 117 (62.8%) | 71 (65.7%) | 46 (59.0%) | 0.12 |
| - - 1 | 72 (37.8%) | 36 (33.3%) | 36 (41.0%) |  |
| - - 2+ | 2 (0.4%) | 1 (1.1%) | 1 (0.9%) |  |
| CCI, median (Q1, Q3) | 4 (3, 4) | 4 (3, 4) | 4 (3, 4) | 0.54 |
| Smoker (including Ex), n (%) | 124 (66.2%) | 76 (70.4%) | 48 (61.5%) | 0.49 |
| Alcohol, n (%) | 86 (46.1%) | 52 (48.1%) | 34 (43.6%) | 0.67 |
| Stage | | | | |
| - - 1 | 71 (38.5%) | 37 (34.3%) | 34 (43.5%) | 0.75 |
| - - 2 | 56 (30%) | 34 (31.5%) | 22(28.2%) |  |
| - - 3 | 44 (23.6%) | 29 (26.9%) | 15 (19%) |  |
| - - 4 | 15 (8%) | 8 (7.4%) | 7 (9.0%) |  |
| Primary cancer site | | | | |
| - Oropharynx | 162 (87%) | 95 (88.0%) | 67(85.9%) | 0.86 |
| - Oral cavity | 2 (1%) | 1 (1.1%) | 1 (1.4%) |  |
| - Larynx | 10(5.3%) | 7 (6.5%) | 3 (3.8%) |  |
| - Hypopharynx/others | 11 (6%) | 5 (4.6%) | 6 (7.7%) |  |
| - - P16+ | 152 (81.7%) | 90 (83.3%) | 62 (79.5%) | 0.47 |
|  |  |  |  |  |
|  |  |  |  |  |
| **Outcomes** | | | | |
| Grade 3 toxicities | 74 (40%) | 42 (38.9%) | 32 (41.0%) | 0.26 |
| Unplanned hospitalization within 30 days of treatment completion | 55 (29.5%) | 34 (31.5%) | 21 (26.6%) | 0.045 |
| Required salvage surgery | 6 (3.2%) | 4 (3.7%) | 2 (2.6) | 0.96 |
| Mortality | 19 (10%) | 13 (12.0%) | 6 (7.7%) | 0.00147 |
| 2-Year TFR (95%-CI) | 8.9% (2.1- 13.1) | 11% (3.2-18.8) | 6.9 (0.1- 10.9) | 0.11 |
| VTE – venous thromboembolic disease, TRAE – treatment-related adverse events, TFR -Treatment Failure Rates | | | | |

**Supplementary Table 3:** Non-Cisplatin-based vs. LD-cisplatin-based therapy

| **Characteristics** | **Overall patients (n=127)** | **Non-Cisplatin  (n=48)** | **LD-Cisplatin  (n=79)** | **p-value** |
| --- | --- | --- | --- | --- |
| **Exposure** | | | | |
| Age (years), median (Q1, Q3) | 62 (56.8, 69.3) | 70 (63, 73.8) | 60 (54.8, 64) | **0.009** |
| Male Sex, n (%) | 109 (85.7%) | 41 (87.5%) | 68 (87.2%) | 0.16 |
| ECOG | | | | |
| - - 0 | 62 (48.5%) | 16 (33.3%) | 46 (591%) | 0.003 |
| - - 1 | 82 (64.5%) | 46 (59%) | 36 (41.0%) |  |
| - - 2+ | 1 (0.9%) | 0 (0%) | 1 (0.9%) |  |
| CCI, median (Q1, Q3) | 4 (3, 5) | 5 (4, 6.8) | 4 (3, 4) | **<0.001** |
| Smoker (including Ex), n (%) | 82 (64.5%) | 34 (70.8%) | 48 (61.5%) | 0.37 |
| Alcohol, n (%) | 56 (44%) | 22 (45.8%) | 34 (43.6%) | 0.79 |
| Stage | | | | |
| - - 1 | 54 (42.5%) | 19 (39.5%) | 35 (34.8%) | 0.58 |
| - - 2 | 42 (33%) | 8 (16.1%) | 34 (31.3%) |  |
| - - 3 | 37 (29%) | 8 (16.1%) | 29 (26.5%) |  |
| - - 4 | 21 (16.5%) | 13 (27.3%) | 8(7.5%) |  |
| Primary cancer site | | | | |
| - Oropharynx | 107 (82.6%) | 38 (72.5%) | 67 (85.5%) | 0.07 |
| - Oral cavity | 2 (1.5%) | 1 (5%) | 1 (1.32%) |  |
| - Larynx | 5 (4%) | 2 (5%) | 3 (3.8%) |  |
| - Hypopharynx/others | 13 (10%) | 7 (17.5%) | 6 (2.7%) |  |
| - - P16+ | 91 (71.6%) | 29 (72.5%) | 62 (79.5.3%) | 0.31 |
| **Outcomes** | | | | |
| Grade 3 toxicities | 58 (45.6%) | 26 (54.5%) | 32 (41.0%) | 0.18 |
| Unplanned hospitalization within 30 days of treatment completion | 33 (25.9%) | 20 (50%) | 13 (17.6%) | **<0.001** |
| Required salvage surgery | 0 (0) | 0 (0) | 0 (0) | **-** |
| Mortality | 15 (12%) | 8 (20%) | 7 (9%) | 0.14 |
| 2-Year TFR (95%-CI) | 7.1 (0.1-14.5) | 8 (0.1 -16.6) | 6.9 (0.2-10.9) | 0.86 |
| VTE – venous thromboembolic disease, TRAE – treatment-related adverse events, TFR -Treatment Failure Rates | | | | |

**Supplementary Table 4:** HD-Cisplatin-based vs. non-cisplatin-based therapy

| **Characteristics** | **Overall patients (n=156)** | **HD-Cisplatin  (n=108)** | **Non-Cisplatin  (n=48)** | **p-value** |
| --- | --- | --- | --- | --- |
| **Exposure** | | | | |
| Age (years), median (Q1, Q3) | 62 (55, 68.3) | 60 (54.8, 64) | 70 (63, 73.8) | **0.009** |
| Male Sex, n (%) | 129 (86.2%) | 96(88.9%) | 33 (82.5%) | 0.42 |
| ECOG | | | | |
| 0 | 81 (52%) | 71 (65.7%) | 10 (25%) | **<0.001** |
| 1 | 65 (42%) | 36 (33.3%) | 29 (72.5%) |  |
| 2+ | 2 (1.2%) | 1 (1.1%) | 1 (2.5%) |  |
| CCI, median (Q1, Q3) | 4 (3, 5) | 4 (3, 4) | 5 (4, 6.8) | **<0.001** |
| Smoker (including Ex), n (%) | 110 (70.5%) | 76 (70.4%) | 34 (70.8%) | 0.77 |
| Alcohol, n (%) | 74 (47.4%) | 52 (48.1%) | 22 (45.7%) | 0.75 |
| Stage | | | | |
| 1 | 56 (35.4%) | 37 (34.3%) | 19(39.6%) | 0.006 |
| 2 | 42 (26.9%) | 34 (31.5%) | 8 (16.7%) |  |
| 3 | 37 (23.7%) | 29 (26.9%) | 8 (16.7%) |  |
| 4 | 21 (13%) | 8 (7.4%) | 13 (27.1%) |  |
|  |  |  |  |  |
| Primary cancer site | | | | |
| - Oropharynx | 133 (85%) | 95 (88.9%) | 38 (72.5%) | 0.38 |
| - Oral cavity | 2 (1.2%) | 1 (1.1%) | 1 (5%) |  |
| - Larynx | 7 (4.4%) | 5 (4.6%) | 2 (4.2%) |  |
| - Hypopharynx/others | 14 (9%) | 7(6.5%) | 7 (14.5%) |  |
| P16+ | 125 (80%) | 90 (83.3%) | 35 (72.9%) | 0.31 |
| **Outcomes** | | | | |
| Grade 3 toxicities | 68 (43.6%) | 42 (38.9%) | 26 (54.5%) | 0.18 |
| Unplanned hospitalization within 30 days of treatment completion | 64 (41.5%) | 34 (37.8%) | 20 (50%) | 0.19 |
| Required salvage surgery | 3 (2%) | 3 (3.3%) | 0 | **-** |
| Mortality | 21 (13) | 13 (14.4%) | 8 (20%) | 0.44 |
| 2-Year TFR (95%-CI) | 7.9 (1.6-17.9) | 9% (3.2-18.8) | 8 (0.1 -16.6) | 0.69 |
| ^#^VTE – venous thromboembolic disease, TRAE – treatment-related adverse events, TFR -Treatment Failure Rates | | | | |

**Supplementary Table 5: Chemotherapy Cumulative Dose, Intensity, and modifications**

|  | **HD Cisplatin** | **LD Cisplatin** | **Carboplatin/paclitaxel** | **Cetuximab** |
| --- | --- | --- | --- | --- |
| No. | **108** | **7** | **30** | **18** |
| Cumulative Dose mg/m^2^, median (Q1, Q3) | 279 (100-300) | 280 (238-280) | - | - |
| Completed Total Prescribed doses | 43 (48%) | 43 (58%) | 12 (50%) | 11 (69%) |
| Dose Reductions | 20(18.5%) | 4 (8.3%) | 3 (12.5%) | 1 (6%) |
| Dose Omissions | 34 (31.5%) | 21 (43.8%) | 14 (58%) | 7 (43.7%) |
| Change to Low-dose | 9 (10%) | - | 0 | - |
| Change to c/p | 10 (11%) | 3 (4%) | - | 0 |
| Switch to Cetuximab | 2 (2%) | 3 (4%) | 0 | - |
| Discontinued permanently | 26 (29%) | 25 (27%) | 12 (50%) | 5 (31%) |

**Supplementary Table 6: Analysis of outcomes in P16+ OP SCC**

|  | **Total** | **HD Cisplatin** | **LD Cisplatin** | **Carboplatin/paclitaxel** | **Cetuximab** |
| --- | --- | --- | --- | --- | --- |
| No. | **188** | **90 (48%)** | **62 (34%)** | **23 (11%)** | **13 (7%)** |
| Stage 1/2 | 144 (76%) | 66 (73%) | 52(82%) | 15 (68%) | 11 (84%) |
| Stage 3/4 | 44 (24%) | 24 (27%) | 11 (16%) | 7 (32%) | 2 (16%) |
| Mortality | 17 (9%) | 8 (9%) | 3 (5%) | 4 (18%) | 2 (15%) |
| 2-Year FFS | 94% | 94.4% | 98% | 82% | 92% |
| Grade 3 Toxicities | 71 (38%) | 35 (38.8%) | 27 (41.2%) | 11 (50%) | 7 (61.5) |
| Unplanned Hospitalization | 63(33.5%) | 28 (31%) | 19 (30%) | 11 (50%) | 5 (38%) |
